# Left ventricular extracellular volume is associated with loss of exercise tolerance in children after Tetralogy of Fallot repair, but not with ventricular dysfunction

**DOI:** 10.1186/1532-429X-17-S1-Q89

**Published:** 2015-02-03

**Authors:** Eugenie Riesenkampff, Wietske Luining, Dariusz Mroczek, Mike Seed, Shi-Joon Yoo, Lars Grosse-Wortmann

**Affiliations:** 1 Hospital for Sick Children, Toronto, ON, Canada

## Background

Diffuse myocardial fibrosis is associated with heart failure and ventricular dysfunction in many diseases. The aim of this study was to relate cardiac magnetic resonance (CMR) measures of diffuse myocardial fibrosis to cardiomechanics and exercise tolerance.

## Methods

In 25 patients (age 13.6±2.5 years, 14 male) after surgical repair of Tetralogy of Fallot (ToF), T1 measurements were performed at a mid and basal ventricular level in short axis before and 15 minutes after the application of 0.1 mmol/kg gadobenate dimeglumine in the interventricular septum (IVS), left ventricular (LV) lateral wall, complete LV myocardium and diaphragmatic portion of the right ventricle (RV). The extracellular volume (ECV) was calculated from native and post-contrast T1 times of myocardium and blood and the hematocrit.

Peak circumferential (LV), longitudinal (RV) and radial (RV) strain as well as the standard deviation of the time to peak strain of different segments (SDpeak) were measured using CMR feature tracking. The correlation of native T1 times and ECV with strain parameters was tested using Pearson's coefficient. Native T1 times and ECV were correlated to exercise test results.

## Results

The mean pulmonary regurgitation fraction was 36±16% and RV enddiastolic volumes were enlarged (EV-EDV 155±52 ml/m^2^) with near-normal ejection fraction (RV-EF 47±8%). LV measures were unremarkable. (Native T1 times and ECV did not differ significantly between different parts of the LV (native T1 time: IVS 1001±52 ms, LV lateral wall 978±55ms, entire LV 982±39ms; ECV: IVS 25±4%, LV lateral wall 23±5%, entire LV 24±3%) and between males and females, but were higher in the RV as compared to the LV (RV native T1 times 1021±69ms (vs. entire LV p<0.05), ECV 29±6% (vs. entire LV p<0.005)). There was no correlation of LV or RV native T1 times and ECV with peak segmental and global strain (global LV peak circumferential strain -22.2±4.6%, RV radial and longitudinal strain of the RV free wall 28.6±18.8% and -11.5±5%) nor with SDpeak (LV 49±30ms or 6.4±3.8% of RR-interval). Exercise test results, available in 11 patients, correlated negatively with ECV of the entire LV (V02 max. r=-0.62, p<0.05, Peak workload r=-0.63, p<0.05).

## Conclusions

In patients after ToF repair, CMR measures of diffuse myocardial fibrosis are associated with exercise intolerance but not with LV or RV dysfunction.

## Funding

This study was partly funded by the Labatt Family Heart Centre, Witchell Fellowship, and by Siemens.

